# Chemosensitivity testing of fresh human gastric cancer with highly purified tumour cells using the MTT assay.

**DOI:** 10.1038/bjc.1992.362

**Published:** 1992-11

**Authors:** H. Yamaue, H. Tanimura, K. Noguchi, T. Hotta, M. Tani, T. Tsunoda, M. Iwahashi, M. Tamai, S. Iwakura

**Affiliations:** Department of Gastroenterological Surgery, Wakayama Medical College, Japan.

## Abstract

A major problem associated with the chemosensitivity testing of fresh human tumour cells using the MTT assay is the contamination of nonmalignant cells in the tumour tissues. Highly purified fresh human gastric cancer cells could be obtained from 43 solid tumours and eight malignant ascites for the MTT assay. The success rate of the MTT assay was 87.9% (51 of the 58 cases), and the purity of tumour cells was greater than 90% after separation on Ficoll-Hypaque and Percoll discontinuous gradients in primary, or metastatic lesions, and also ascites. Cisplatin, mitomycin, and doxorubicin were more potent drugs than etoposide and 5-FU against gastric cancer cells. The chemosensitivity in differentiated cancer was equivalent to that in non-differentiated cancer. Twenty of the 51 patients with gastric cancer had evaluable lesions, and they received chemotherapy according to the results of the MTT assay using highly purified tumour cells. A clinical response was obtained in 12 of these 20 patients (response rate: 60.0%; five with complete response, seven with partial response).


Br. J. Cancer (1992), 66, 794–799 © Macmillan Press Ltd., 1992

### Chemosensitivity testing of fresh human gastric cancer with highly purified tumour cells using the MTT assay

H. Yamaue, H. Tanimura, K. Noguchi, T. Hotta, M. Tani, T. Tsunoda, M. Iwahashi, M. Tamai & S. Iwakura

*Department of Gastroenterological Surgery, Wakayama Medical College, 27-Shichibancho, Wakayama 640, Japan.*

**Summary** A major problem associated with the chemosensitivity testing of fresh human tumour cells using the MTT assay is the contamination of nonmalignant cells in the tumour tissues. Highly purified fresh human gastric cancer cells could be obtained from 43 solid tumours and eight malignant ascites for the MTT assay. The success rate of the MTT assay was 87.9% (51 of the 58 cases), and the purity of tumour cells was greater than 90% after separation on Ficoll-Hypaque and Percoll discontinuous gradients in primary, or metastatic lesions, and also ascites. Cisplatin, mitomycin, and doxorubicin were more potent drugs than etoposide and 5-FU against gastric cancer cells. The chemosensitivity in differentiated cancer was equivalent to that in non-differentiated cancer. Twenty of the 51 patients with gastric cancer had evaluable lesions, and they received chemotherapy according to the results of the MTT assay using highly purified tumour cells. A clinical response was obtained in 12 of these 20 patients (response rate: 60.0%; five with complete response, seven with partial response).

A rapid colorimetric assay was described by Mosmann (1983) for determining the ability of viable cells to convert a soluble tetrazolium salt, 3-(4,5-di-methylthiazol-2-yl)-2,5-diphenyl tetrazolium bromide (MTT), into an insoluble formazan precipitate. The MTT assay is a rapid and quantitative colorimetric system for the determination of the chemosensitivity of tumour cell lines (Carmichael *et al.*, 1987; Park *et al.*, 1987; Schroyens *et al.*, 1990), and also in fresh leukaemia cells (Twentyman *et al.*, 1989; Pieters *et al.*, 1989; Hanson *et al.*, 1991). However, the use of this assay for solid tumour tissues has been limited because of contamination by nonmalignant cells in tumour specimens (Kaspers *et al.*, 1991; Campling *et al.*, 1991; Suto, 1991). Thus, when the MTT assay is employed for chemosensitivity testing of tumour samples, highly purified fresh tumour cells should be used, because contamination by nonmalignant cells affects the results of this assay (Yamaue *et al.*, 1991; Campling *et al.*, 1991; Suto, 1991).

The present study was designed to determine the chemosensitivity in fresh human gastric cancer, using highly purified tumour cells, and the correlation of this sensitivity with clinical response.

### Patients and methods

Fifty-eight patients with gastric cancer were entered in this study. Tumour specimens and ascites were taken for diagnostic or therapeutic indications, and the informed consent of the patients was obtained for the use of samples for drug sensitivity testing. The MTT assay could be performed in 51 of the 58 patients (success rate: 87.9%). The reasons for the seven unsuccessful assays were: four with low optical density (OD57); less than 0.1 after culture, two with few viable cells due to tumour necrosis, and one with bacterial contamination during culture. Surgical specimens were obtained from 43 of the 51 patients; 33 patients had primary gastric lesions, eight had metastatic lymph nodes, one had liver metastasis, and one had ovary metastasis. Peritoneal effusions were collected for analysis from eight patients with disseminated gastric cancer. The clinical stages of the 51 patients according to the TNM classification of malignant tumours by UICC were: four with Stage II, 12 with Stage IIIB, and 35 with Stage IV.

None of these patients had received any previous antitumour drugs.

### *Antitumour drugs*

The antitumour drugs tested were cisplatin (CDDP), etoposide (VP-16), mitomycin C (MMC), doxorubicin (DOX), and 5-fluorouracil (5-FU). Each drug was diluted in complete medium at therapeutic peak plasma concentration (Cmax × 1) achieved by intravenous administration of clinical doses (Scheithauer *et al.*, 1986). The values were: MMC 1.0 $\mu g\ ml^{-1}$; 5-FU 10 $\mu g\ ml^{-1}$; DOX 0.4 $\mu g\ ml^{-1}$; CDDP 2.0 $\mu g\ ml^{-1}$; and VP-16 10 $\mu g\ ml^{-1}$, and 10-fold equivalents were also prepared (Cmax × 10). The complete medium used consisted of RPMI-1640 (Nissui Co., Tokyo, Japan) supplemented with 10% heat-inactivated foetal calf serum (GIBCO, New York, USA), 2 mM L-glutamine, and antibiotics (100 U penicillin $ml^{-1}$ and 100 $\mu g$ streptomycin $ml^{-1}$).

### *Purification of fresh human gastric cancer cells*

Malignant ascites was immediately centrifuged at 400 *g* for 5 min and then suspended in complete medium. Freshly excised tumour tissues were processed using enzymatic digestion, as previously described (Yamaue *et al.*, 1990*a*). Briefly, tumour tissues were dissected into pieces smaller than 2 $mm^3$ which were immersed in complete medium containing collagenase (2 $mg\ ml^{-1}$, type V-S; Sigma), hyaluronidase (10 units $ml^{-1}$, type IV-S; Sigma), and DNase-I (0.4 $mg\ ml^{-1}$; Sigma). After 40 min incubation at 37°C, the cells were harvested, washed, and suspended in complete medium.

The purification of autologous tumour cells has also been previously described (Yamaue *et al.*, 1990*b*; 1991). Tumour cells obtained from solid tumour specimens and ascites were centrifuged on Ficoll-Hypaque (specific gravity 1.077; Pharmacia, Uppsala, Sweden) gradients at 400 *g* for 30 min in 50 ml tubes. The interface was collected, and suspended at a concentration of $1 \times 10^6\ ml^{-1}$ in complete medium. The cells were then layered on discontinuous gradients consisting of 10 ml of 100% and 15 ml of 75% Ficoll-Hypaque in 50 ml plastic tubes. After centrifugation at 400 *g* for 30 min, a tumour cell-rich fraction was collected from the 75% interface. The tumour cell-enriched suspension was then layered

Correspondence: H. Yamaue, Department of Gastroenterological Surgery, Wakayama Medical College, 27-Shichibancho, Wakayama 640, Japan.
Received 17 February 1992; and in revised form 1 July 1992.

onto discontinuous gradients containing 4 ml each of 25%, 15%, and 10% Percoll (Pharmacia, Uppsala, Sweden) in complete medium in 15 ml plastic tubes. Centrifugation was performed at 25 *g* for 7 min, and tumour cells depleted of lymphoid cells were collected from the bottom and from the 25% interface, and suspended in complete medium at a concentration of $1 \times 10^6\ ml^{-1}$. The cells thus prepared were primarily tumour cells, with less than 10% contamination by nonmalignant cells, as judged by morphologic examination using Papanicolaou staining or carcinoembryonic antigen (CEA) staining for CEA-positive tumour cells. The cells were found to be more than 90–95% viable by the trypan blue dye exclusion test. The nonmalignant cells including tumour-infiltrating lymphocytes, fibroblasts, and mesothelial cells, were removed by the purification procedures. The mean yield of purified tumour cells was $2.5 \pm 0.7 \times 10^6$, and the tumour cell count at the beginning of preparation was $14 \pm 4.3 \times 10^6$ (rate of yield: 17.9%)

### *Method of MTT assay*

Chemosensitivity was assessed using the tetrazolium salt MTT (Sigma No. M2128) to measure the viability of tumour cells (Mosmann, 1983; Yamaue *et al.*, 1991). One hundred μl of tumour cells suspension ($1 \times 10^6$ cells $ml^{-1}$) was added to 25 μl of each drug at final concentration of Cmax × 10 and Cmax × 1, in 96-well flat-bottomed microtitre plates (Corning No. 25860), and incubated at 37°C in a humidified 5% $CO_2$ atmosphere for 96 h. The chemosensitivity assay was assessed in triplicate. Three microtitre wells containing tumour cells suspended in 125 μl of complete medium (total tumour cell number was equivalent to that in the test wells) were used as controls for cell viability, and three wells containing only complete medium were used as controls for nonspecific dye reduction. After incubation, the plates were centrifuged, the supernatants were removed, and 30 μl/well of MTT solution with 10 μM of sodium succinate was added to all the wells. The plates were incubated for an additional 4 h, and 150 μl of dimethyl sulfoxide (DMSO) was then added to all the wells (Carmichael *et al.*, 1987); the mixtures were pipetted thoroughly to dissolve the dark blue crystals. The plates were then read on a microplate reader (Corona Electric, MTP-32) using a test wavelength of 570 nm and a reference wavelength of 630 nm. The control wells without tumour cells had an OD of less than 0.005, and the samples in which the OD was over 0.1 were accepted for the assay.

The inhibition rate was calculated as follows:

Inhibition rate = (1 – OD drug treated/OD control) × 100

The background of tumour cells (including dead cells) without addition of MTT had an OD of less than 0.012 after 96 h incubation, and the influence of dead tumour cells could therefore be ignored in the present study. The viability of tumour cells was maintained at 75–90% during the 96 h incubation, and the $OD_{570}$ values before and after 96 h incubation were $0.36 \pm 0.17$, and $0.33 \pm 0.14$, respectively.

### *Chemotherapy on the basis of the results of the MTT assay*

Twenty of the 51 patients whose tumour cells could be assayed had evaluable lesions. These patients received cancer chemotherapy on the basis of the results of the MTT assay. Two or three drugs were administered, of which inhibition rates were generally more than 50% at ten times the peak plasma concentration. The disease stages in the remaining 31 patients were; four with Stage II; 12 with Stage IIIB, and 15 with Stage IV. The patients with Stage II and IIIB received Tegafur (FT-207) orally as adjuvant chemotherapy following curative operation. The patients with Stage IV were: ten with T4N2M0 and five with M1 (LYM), and they also received cancer chemotherapy on the basis of the results of the MTT assay. However, no evaluation could be done, since there were no evaluable lesions after surgery.

Informed consent for the studies was obtained from all subjects, in accordance with the guidelines of the Ethical Committee on Human Research, Wakayama Medical College.

### *Statistical analysis*

Significant differences were determined by paired *t*-tests or nonparametric Wilcoxon signed-rank test, and the generalised Wilcoxon test was used for survival. A *P* value of less than 0.05 was considered to be statistically significant.

### Results

### *Purity of fresh human gastric cancer cells*

The purity of tumour cells before and immediately after enzymatic digestion alone for primary tumours and lymph nodes, or centrifugation alone for malignant ascites was 43.6 ± 14.1%, 40.8 ± 16.8% and 53.5 ± 23.8% respectively. The purity after processing on the Ficoll-Hypaque discontinous gradients increased to 63.9 ± 12.4%, 68.1 ± 13.2% and 62.7 ± 17.1%, respectively. Tumour cells in solid tumours, including primary and metastatic lymph nodes, and in malignant ascites samples, were enriched to 90% using the Percoll discontinuous gradients ($P < 0.01$) (Table I).

### *Chemosensitivity of purified gastric tumour cells*

At a drug concentration of Cmax × 10, the inhibition rates of tumour cells for CDDP were higher than those for VP-16, 5-FU ($P < 0.01$), and for DOX ($P < 0.05$). However at Cmax × 1, the inhibition rates for CDDP, MMC, DOX, and 5-FU were higher than that for VP-16 (Table II).

### *Comparison of chemosensitivity between well and poorly differentiated gastric cancer*

Pathological examinations of primary gastric lesions showed that 18 were the well differentiated (papillary and tubular adenocarcinomas) and 15 were the poorly differentiated, including six with signet-ring cell carcinoma and one with mucinous adenocarcinoma.

As shown in Figure 1, there were no significant differences between the chemosensitivity of the well differentiated and the poorly differentiated type at drug concentrations of Cmax × 10 and Cmax × 1. However, the inhibition rates were slightly higher for the poorly differentiated tissues than for the well differentiated tissues when exposed to MMC, DOX, and 5-FU at Cmax × 10, and DOX and 5-FU at Cmax × 1 ($0.05 < P < 0.1$) (Figure 1).

### *Clinical correlation*

Of the 51 patients, 20 had evaluable lesions; and they received cancer chemotherapy according to the results of the MTT assay using highly purified tumour cells. Clinical res-

**Table I** Purity of tumour cells

| Preparation step | Primary tumour $n = 33$ | Lymph nodes $n = 8$ | Ascites $n = 8$ |
|---|---|---|---|
| | Purity (%) (Absolute cell numbers: $\times 10^6$) | | |
| Before purification | 43.6 ± 14.1<br>(31.5 ± 9.9) | 40.8 ± 16.8<br>(30.7 ± 11.1) | 53.5 ± 23.8<br>(37.3 ± 14.8) |
| After purification | 92.2 ± 5.3[a]<br>(2.7 ± 0.8[a]) | 90.4 ± 4.4[a]<br>(2.6 ± 0.9[a]) | 91.8 ± 3.9[a]<br>(2.8 ± 0.8[a]) |

The tumour cells were enriched to a purity of 90% after purification. [a]$P < 0.01$, compared with the purity or absolute cell numbers obtained by enzymatic digestion or centrifugation alone. The absolute cell numbers including tumour cells and nonmalignant cells decreased from $31.5 \times 10^6$ to $2.7 \times 10^6$ after purification in primary tumour.

**Table II** Chemosensitivity of purified gastric cancer cells

| *Concentration* | *Inhibition rates (%) by anticancer agents* | | | | |
|---|---|---|---|---|---|
| | *CDDP* | *VP-16* | *MMC* | *DOX* | *5-FU* |
| Cmax × 10 ($n = 51$) | 66.1±28.4[a] | 42.9±23.5[b] | 63.1±26.3 | 54.9±30.2 | 49.0±26.2 |
| Cmax × 1 ($n = 32$) | 29.6±15.4[c] | 11.6±11.7[d] | 27.6±23.9 | 27.0±24.4 | 33.1±27.1 |

[a]$P < 0.01$, compared with the inhibition rates of VP-16 and 5-FU; $P < 0.05$, compared with that of DOX at Cmax × 10. [b]$P < 0.01$, compared with that of MMC; $P < 0.05$, compared with that of DOX. [c]$P < 0.01$, compared with the inhibition rate of VP-16 at Cmax × 1. [d]$P < 0.01$, compared with that of MMC, DOX, and 5-FU. CDDP = cisplatin, VP-16 = etoposide, MMC = mitomycin, DOX = doxorubicin, 5-FU = 5-fluorouracil.

**Figure 1** Comparison of chemosensitivity in well and poorly differentiated gastric cancer. The chemosensitivity of gastric cancer with poorly differentiated type was equivalent to that of the well differentiated type not only at Cmax × 10, but also at Cmax × 1.

ponses were obtained in 12 of the 20 patients (response rate: 60.0%). Of the five patients showing complete response (CR), the lesions in two patients were metastatic lymph nodes, including cervical lymph nodes and para-aortic lymph nodes. Seven patients had a partial response (PR), of which the evaluable lesions were three with malignant ascites, three with metastatic lymph nodes, and one with abdominal tumour (Table III).

Table IV shows the clinical outcomes of the nonresponders to chemotherapy administered according to the MTT assay results. Seven patients showed no change (NC); the lesions in these patients were metastatic lymph nodes in four, and malignant ascites in three. One patient with liver metastasis had progressive disease (PD), and her survival was 2.6 months. The mean survival of responders ($n = 12$) was 264.4 days, and that of nonresponders ($n = 8$) was only 134.6 days ($P = 0.013$, by generalised Wilcoxon test). The mean survival of five patients with M1 (LYM) among the 31 patients in whom the lesions could not be evaluated was 192.0 days. There was no difference in doses of anticancer drugs administered to patients who had a clinical response and those who did not.

*Comparison of chemosensitivity in patients with and without clinical response*

The inhibition rates for CDDP and MMC were significantly higher ($P < 0.05$) in patients with clinical responses who had received chemotherapy based on the results of the MTT assay, than in those without clinical responses, whereas the chemosensitivity for VP-16, DOX and 5-FU was equivalent in the two groups (Table V).

### Discussion

The MTT assay for chemosensitivity testing is a rapid and semi-automated quantitative assay for screening the effects of anticancer drugs on fresh tumour samples (Twentyman *et al.*, 1989; Wilson *et al.*, 1990; Yamaue *et al.*, 1991), as well as on established cell lines (Twentyman *et al.*, 1987; Carmichael *et al.*, 1987; Park *et al.*, 1987). However, contamination by non-malignant cells in tumour tissues influences the results of this assay, although the nonmalignant cells are less sensitive to anticancer drugs than the malignant cells (Maehara *et al.*, 1989; Yamaue *et al.*, 1991; Kaspers *et al.*, 1991). To use this assay for leukaemic cells in peripheral blood or bone marrow, the cells must be enriched to more than 90% by centrifugation with 100% Ficoll-Hypaque alone (Sargent & Taylor, 1989).

The MTT assay has been performed using malignant cells from solid tumour samples, including lung cancer (Campling *et al.*, 1991) and ovarian cancer (Wilson *et al.*, 1990) prepared with 100% Ficoll-Hypaque alone, or using malignant cells from gastrointestinal cancer tissues prepared by enzymatic digestion alone (Yamauchi *et al.*, 1991). The purity of malignant cells from ovarian cancer has been reported to be relatively high; i.e. 70–80% (Wilson *et al.*, 1990), however, there is marked contamination by nonmalignant cells in gastric cancer tissues as shown in the present study.

Thus, the MTT assay should be performed with highly purified tumour cells in gastric cancer, since the chemosensitivity of malignant tumour cells is distinct from that of non-malignant cells, and moreover, since the chemosensitivity of highly purified tumour cells is also distinct from that of non-purified cells merely separated from tumour tissues (Yamaue *et al.*, 1991).

Gastrointestinal cancer is more resistant to anticancer drugs than leukaemia and malignant lymphoma (Yamauchi *et al.*, 1991), and gastric adenocarcinoma is less sensitive to these drugs than lung adenocarcinoma (Kohnoe *et al.*, 1991). In the present study, the inhibition rates of fresh human gastric cancer cells with CDDP, MMC, DOX, and 5-FU were 66.1%, 63.1%, 54.9%, and 49.0%, respectively, whereas they were 57.9%, 49.3%, 40.6% and 26.4% in a study by Kohnoe *et al.* (1991). The effective rates for CDDP, VP-16, MMC, and DOX were 45.5%, 10.0%, 66.7% and 41.7%, respectively (Ohyama *et al.*, 1991) (all data at Cmax × 10). The differences in these findings may be related to the purification of the tumour cells. While Maehara *et al.* (1987) found that the inhibition rates on the MTT assay were remarkably high for poorly differentiated gastric cancer tissues, exposed to CDDP, MMC, DOX, and 5-FU, compared with those for well differentiated tissues. We found no significant differences between the chemosensitivity of these two types of gastric cancer tissues.

**Table III** Clinical response according to the MTT assay

| *Case* | *Age/Sex* | *Stage* | *Evaluable lesion* | *Chemotherapy (mean ± s.d.ª)* | | | *Response* | *Survival (mo)* |
|---|---|---|---|---|---|---|---|---|
| 19 | 68/F | IV | Lymph nodes | CDDP, (60±2) | VP-16 (47±2) | | CR | 9.7 |
| 9 | 39/F | IV | Malignant ascites | CDDP, (68±3) | VP-16 (14±1) | MMC (55±2) | CR | 9.5 |
| 16 | 56/F | IV | Malignant ascites | MMC, (88±3) | DOX, (71±2) | 5-FU (76±3) | CR | 9.5 |
| 26 | 45/F | IV | Malignant ascites | CDDP, (66±3) | VP-16 (71±2) | | CR | 10.9 (alive) |
| 46 | 28/F | IV | Lymph nodes | CDDP (100±3) | | | CR | 6.3 (alive) |
| 21 | 62/M | IV | Abdominal tumour | MMC, (89±3) | DOX, (100±4) | 5-FU (56±2) | PR | 13.8 |
| 5 | 56/M | IV | Malignant ascites | MMC, (98±4) | 5-FU (43±1) | | PR | 13.5 |
| 2 | 39/F | IV | Malignant ascites | CDDP, (99±3) | MMC (92±4) | | PR | 12.0 |
| 11 | 66/F | IV | Malignant ascites | CDDP, (100±3) | MMC (50±2) | | PR | 5.0 |
| 29 | 52/M | IV | Lymph nodes | CDDP, (84±5) | VP-16 (46±2) | | PR | 3.9 |
| 25 | 59/F | IV | Lymph nodes | CDDP, (65±2) | VP-16, (72±4) | DOX (45±1) | PR | 3.5 |
| 40 | 51/M | IV | Lymph nodes | CDDP, (86±2) | MMC (51±3) | | PR | 6.7 (alive) |

Cisplatin and mitomycin were administered intraperitoneally for malignant ascites, and other drugs were given intravenously. The absorbance values in OD570 of control wells with tumour cells alone were as follows; Case 19 = 0.53, Case 9 = 0.17, Case 16 = 0.40, Case 26 = 0.25, Case 46 = 0.28, Case 21 = 0.39, Case 5 = 0.33, Case 2 = 0.52, Case 11 = 0.75, Case 29 = 0.37, Case 25 = 0.37, and Case 40 = 0.29. [a]Mean ± s.d. of the inhibition rates (%) in triplicate wells at Cmax × 10.

**Table IV** Nonresponders to chemotherapy given according to the MTT assay

| *Case* | *Age/Sex* | *Stage* | *Evaluable lesion* | *Chemotherapy (mean ± s.d.ª)* | | | *Response* | *Survival (mo)* |
|---|---|---|---|---|---|---|---|---|
| 17 | 48/M | IV | Lymph nodes | CDDP, (67±3) | DOX, (36±1) | 5-FU (65±2) | NC | 9.8 |
| 35 | 63/M | IV | Lymph nodes | MMC, (31±1) | DOX, (48±2) | 5-FU (65±2) | NC | 7.4 |
| 13 | 65/F | IV | Malignant ascites | CDDP, (76±3) | VP-16, (58±2) | MMC (61±2) | NC | 4.7 |
| 3 | 51/F | IV | Malignant ascites | CDDP, (67±3) | VP-16, (31±1) | MMC (34±2) | NC | 3.5 |
| 7 | 58/M | IV | Lymph nodes | CDDP, (72±3) | MMC, (60±2) | DOX (61±2) | NC | 3.3 |
| 4 | 57/M | IV | Malignant ascites | CDDP, (55±4) | VP-16, (18±1) | 5-FU (50±2) | NC | 2.4 |
| 10 | 79/F | IV | Lymph nodes | MMC, (60±3) | DOX, (38±1) | 5-FU (47±2) | NC | 2.2 |
| 8 | 63/F | IV | Liver metastasis | CDDP, (45±1) | VP-16, (68±3) | MMC (64±2) | PD | 2.6 |

The absorbance values in OD570 of control wells with tumour cells alone were as follows: Case 17 = 0.15, Case 35 = 0.25, Case 13 = 0.36, Case 3 = 0.61, Case 7 = 0.35, Case 4 = 0.43, Case 10 = 0.34, Case 8 = 0.28. [a]Mean ± s.d. of the inhibition rates (%) in triplicate wells at Cmax × 10.

Another problem associated with the clinical application of the MTT assay is the determination of optimal conditions for the evaluation of chemosensitivity and drug concentration. Camling *et al.* (1991) and Schroyens *et al.* (1990) reported that the data for the MTT assay in established cell lines should be expressed as the area under the dose response curves (AUC); however, the usefulness of the MTT assay in clinical samples may be of limited value, since adequate numbers of tumour cells, to enable the assessment of the AUC for several drugs, cannot always be obtained in all cases. Therefore, we used two drug concentrations, including Cmax × 10 and Cmax × 1, for the MTT assay of clinical samples; however, Cmax levels of drugs will vary according to the method of measurement, the clinical protocol, and the individual patient. Many other factors are also involved in drug activity, including alterations to drug metabolites.

In chemosensitive leukaemia, since the lethal concentration to 50% of the cells ($LD_{50}$) in the dose-response curve was found to be equivalent to Cmax × 1, drugs induced 50% cytotoxicity were considered to be effective at Cmax × 1 (Kaspers *et al.*, 1991). In an MTT assay for ovarian cancer, Wilson *et al.* (1990) employed the criteria used for predicting *in vivo* sensitivity in haematological malignancy, in which the drug concentration was equivalent to Cmax × 2 (Weisenthal *et al.*, 1986). On the other hand, in human colo-rectal carcinoma cell lines, the AUC which produced 50% growth inhibition was within a clinically achievable range (Cmax × 1) only for 5-FU (Park *et al.*, 1987), and Cmax × 10-100 was required to reduce 50% of the AUC for other drugs, including CDDP, VP-16, MMC and DOX. These findings are supported by our results that the inhibition rates of 5-FU were 49.0% at Cmax × 10, and 33.1% at Cmax × 1, and that the difference between Cmax × 10 and Cmax × 1 was minimal. In fresh human gastrointestinal cancer, the concentra-

Table V Comparison of chemosensitivity in patients with and without clinical response

| | Inhibition rates (%) by anticancer agents | | | | |
|---|---|---|---|---|---|
| | CDDP | VP-16 | MMC | DOX | 5-FU |
| Response (+) ($n = 12$) | 80.9±15.5[a] | 49.9±21.1 | 74.7±19.9[a] | 72.1±22.5 | 57.3±14.0 |
| Response (−) ($n = 8$) | 63.5±10.7 | 43.9±20.0 | 51.6±12.5 | 45.7±9.97 | 54.1±7.85 |

[a]$P < 0.05$, compared to nonresponders.

tion of drugs used in the MTT assay has usually been Cmax × 10 (Maehara *et al.*, 1987; Yamaue *et al.*, 1991; Ohyama *et al.*, 1991). In the present study, the inhibition rates obtained with anticancer drugs ranged from 11.6% (VP-16) to 33.1% (5-FU) at Cmax × 1, and the evaluation could be performed. Therefore, the results obtained at Cmax × 1 should be considered to be clinically applicable.

In retrospective studies, the MTT assay has accurately predicted the initial response to chemotherapy in acute leukaemia (Sargent & Taylor, 1989; Santini *et al.*, 1989), as well as the long-term clinical outcome (Pieters *et al.*, 1991). The present study was prospective, being designed to determine chemotherapy according to the results of the MTT assay. Clinical response rate was obtained in 12 of the 20 patients (60.0%). We consider this rate to be relatively high, since the response rate for conventional chemotherapy in gastric cancer in our hospital was only 15.9% (Yamaue *et al.*, 1990*c*). However, a prospective randomised-controlled study with an adequate number of patients is required for the evaluation of whether the results of the MTT assay *in vitro* correlate with the clinical response *in vivo*.

Our investigations continue, by a randomised-controlled prospective study with adequate number of patients, to examine whether the results of the MTT assay using highly purified fresh human tumour cells correlate with clinical response, and further, which drug concentrations should be used in the MTT assay.

### References

CAMPLING, B.J., PYM, J., BAKER, H.M., COLE, S.P.C. & LAM, Y.M. (1991). Chemosensitivity testing of small cell lung cancer using the MTT assay. *Br. J. Cancer*, **63**, 75–83.

CARMICHAEL, J., DEGRAFF, W.G., GAZDAR, A.F., MINNA, J.D. & MITCHELL, J.B. (1987). Evaluation of a tetrazolium-based semiautomated colorimetric assay: assessment of chemosensitivity testing. *Cancer Res.*, **47**, 936–942.

HANSON, J.A., BENTLEY, D.P., BEAN, E.A., NUTE, S.R. & MOORE, J.L. (1991). *In vitro* chemosensitivity testing in chronic lymphocytic leukaemia patients. *Leukemia Res.*, **15**, 565–569.

KASPERS, G.J.L., PIETERS, R., VAN ZANTWIJK, C.H., DELAAT, P.A.J.M., DEWAAL, F.C, VAN WERING, E.R. & VEERMAN, A.J.P. (1991). *In vitro* drug sensitivity of normal peripheral blood lymphocytes and childhood leukaemia cells from bone marrow and peripheral blood. *Br. J. Cancer*, **64**, 469–474.

KOHNOE, S., MORIGUCHI, S., EMI, Y., SAKAGUCHI, Y., MAEHARA, Y., ISHIDA, T., MITSUDOMI, T. & SUGIMACHI, K. (1991). Lung adenocarcinoma is more sensitive than gastric adenocarcinoma to anticancer drugs *in vitro*. *Eur. J. Surg. Oncol.*, **17**, 47–50.

MAEHARA, Y., ANAI, H., KUSUMOTO, H. & SUGIMACHI, K. (1987). Poorly differentiated human gastric carcinoma is more sensitive to antitumor drugs than is well differentiated carcinoma. *Eur. J. Surg. Oncol.*, **13**, 203–206.

MAEHARA, Y., KUSUMOTO, H., KUSUMOTO, T., ANAI, H. & SUGIMACHI, K. (1989). Tumor tissue is more sensitive to mitomycin C, carboquone, and aclacinomycin A than is adjacent normal tissue *in vitro*. *J. Surg. Oncol.*, **40**, 4–7.

MOSMANN, T. (1983). Rapid colorimetric assay for cellular growth and survival: application to proliferation and cytotoxicity assays. *J. Immunol. Methods*, **65**, 55–63.

OHYAMA, S., TANAKA, M., YONEMURA, Y., KINOSHITA, K., MIYAZAKI, I. & SASAKI, T. (1991). *In vitro* chemosensitivity test of human gastric carcinomas using collagen gel matrix. *Jpn. J. Cancer Res.*, **82**, 607–612.

PARK, J.-G., KRAMER, B.S., STEINBERG, S.M., CARMICHAEL, J. & COLLINS, J.M. (1987). Chemosensitivity testing of human colorectal carcinoma cell lines using a tetrazolium-based colorimetric assay. *Cancer Res.*, **47**, 5875–5879.

PIETERS, R., HUISMANS, D.R., LEYVA, A. & VEERMAN, A.J.P. (1989). Comparison of the rapid automated MTT-assay with a dye exclusion assay for chemosensitivity testing in childhood leukaemia. *Br. J. Cancer*, **59**, 217–220.

PIETERS, R., HUISMANS, D.R., LOONEN, A.H., HAHLEN, K., VAN DER DOES-VAN DEN BERG, A., VAN WERING, E.R. & VEERMAN, A.J.P. (1991). Relation of cellular drug resistance to long-term clinical outcome in childhood acute lymphoblastic leukaemia. *Lancet*, **338**, 399–403.

SANTINI, V., BERNABEI, P.A., SILVESTRO, L., POZZO, O.D., BEZZINI, R., VIANO, I., GATTEI, V., SACCARDI, R. & FERRINI, P.R. (1989). *In vitro* chemosensitivity testing of leukemic cells: prediction of response to chemotherapy in patients with acute non-lymphocytic leukaemia. *Hematol. Oncol.*, **7**, 287–293.

SARGENT, J.M. & TAYLOR, C.G. (1989). Appraisal of the MTT assay as a rapid test of chemosensitivity in acute myeloid leukaemia. *Br. J. Cancer*, **60**, 206–210.

SCHEITHAUER, W., CLARK, G.M., SALMON, S.E., DORDA, W., SHOEMAKER, R.H. & VON HOFF, D.D. (1986). Model for estimation of clinically achievable plasma concentrations for investigational anticancer drugs in man. *Cancer Treat. Rep.*, **70**, 1379–1382.

SCHROYENS, W., TUENI, E., DODION, P., BODECKER, R., STOESSEL, F. & KLASTERSKY, J. (1990). Validation of clinical predictive value of *in vitro* colorimetric chemosensitivity assay in head and neck cancer. *Eur. J. Cancer*, **26**, 834–838.

SUTO, A. (1991). The influence of stromal cells on the MTT assay I. *In vitro* chemosensitivity of the tumor and stromal cells to mitomycin C. *Jpn. J. Surg.*, **21**, 304–307.

TWENTYMAN, P.R. & LUSCOMBE, M. (1987). A study of some variables in a tetrazolium dye (MTT) based assay for cell growth and chemosensitivity. *Br. J. Cancer*, **56**, 279–285.

TWENTYMAN, P.R., FOX, N.E. & REES, J.K.H. (1989). Chemosensitivity testing of fresh leukaemia cells using the MTT colorimetric assay. *Br. J. Haematol.*, **71**, 19–24.

WEISENTHAL, L.M., DILL, P.L., FINKLESTEIN, J.Z., DUARTE, T.E., BAKER, J.A. & MORAN, E.M. (1986). Laboratory detection of primary and acquired drug resistance in human lymphatic neoplasms. *Cancer Treat. Rep.*, **70**, 1283–1295.

WILSON, J.K., SARGENT, J.M., ELGIE, A.W., HILL, J.G. & TAYLOR, C.G. (1990). A feasibility study of the MTT assay for chemosensitivity testing in ovarian malignancy. *Br. J. Cancer*, **62**, 189–194.

YAMAUCHI, M., SATTA, T., ITO, A., KONDO, T. & TAKAGI, H. (1991). A feasibility study of the SDI test for the evaluation of gastrointestinal cancer sensitivity to anticancer drugs. *J. Surg. Oncol.*, **47**, 253–260.

YAMAUE, H., TANIMURA, H., TANI, M., IWAHASHI, M., TSUNODA, T. & INOUE, M. (1990*a*). *In vitro* antitumor activity of a new platinum analogue, NK121 against fresh human tumor cells and established tumor cell lines by succinate dehydrogenase inhibition test. *Chemotherapy (Tokyo)*, **38**, 780–789.

YAMAUE, H., TANIMURA, H., TSUNODA, T., IWAHASHI, M., TANI, M., TAMAI, M. & INOUE, M. (1990*b*). Functional and phenotypic analyses of interleukin 2-activated tumor-infiltrating lymphocytes. *Biotherapy*, **2**, 247–259.

YAMAUE, H., TANIMURA, H., TERASHITA, S., IWAHASHI, M., TANI, M., TSUNODA, T., TAMAI, M. & MORI, K. (1990*c*). Clinical evaluation of chemotherapy under angiotensin II-induced hypertension in patients with advanced cancer. *Arch. Jpn. Chir.*, **59**, 302-309.

YAMAUE, H., TANIMURA, H., TSUNODA, T., TANI, M., IWAHASHI, M., NOGUCHI, K., TAMAI, M., HOTTA, T. & ARII, K. (1991). Chemosensitivity testing with highly purified tumour cells with the MTT colorimetric assay. *Eur. J. Cancer*, **27**, 1258-1263.


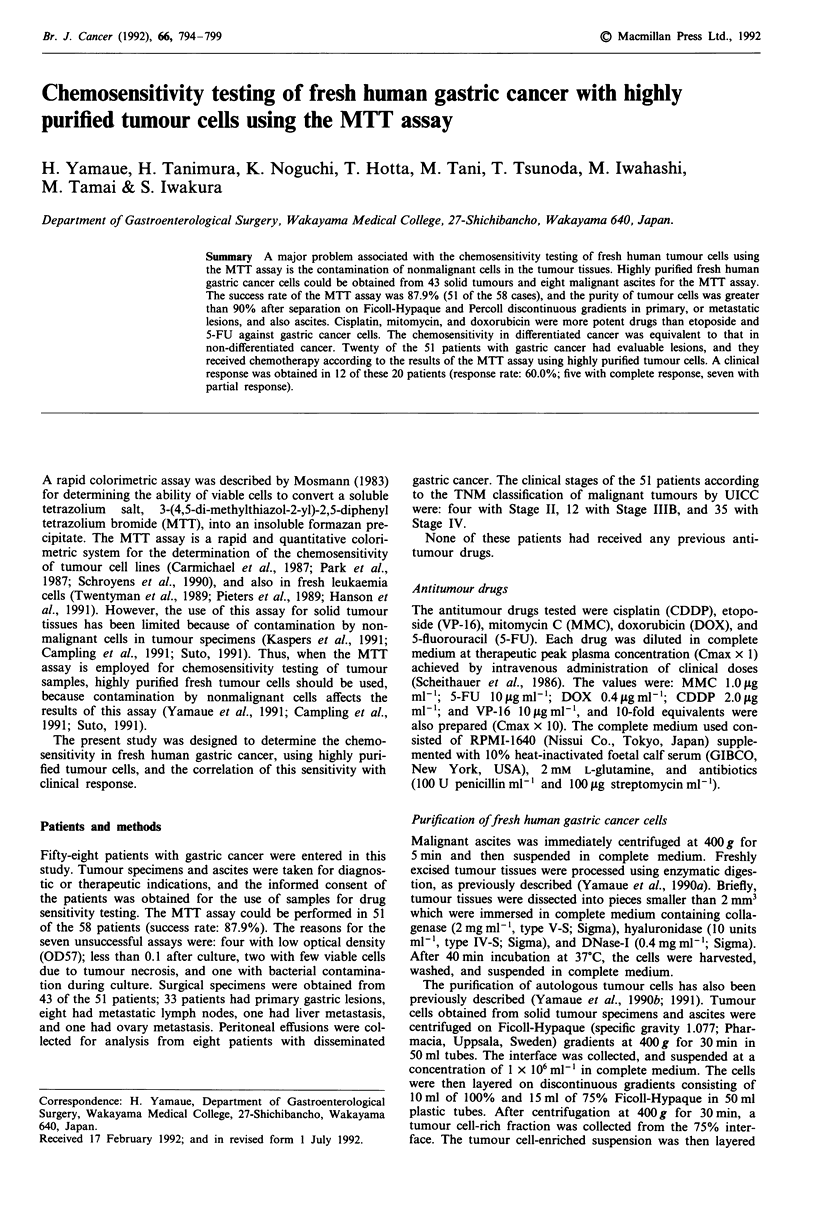

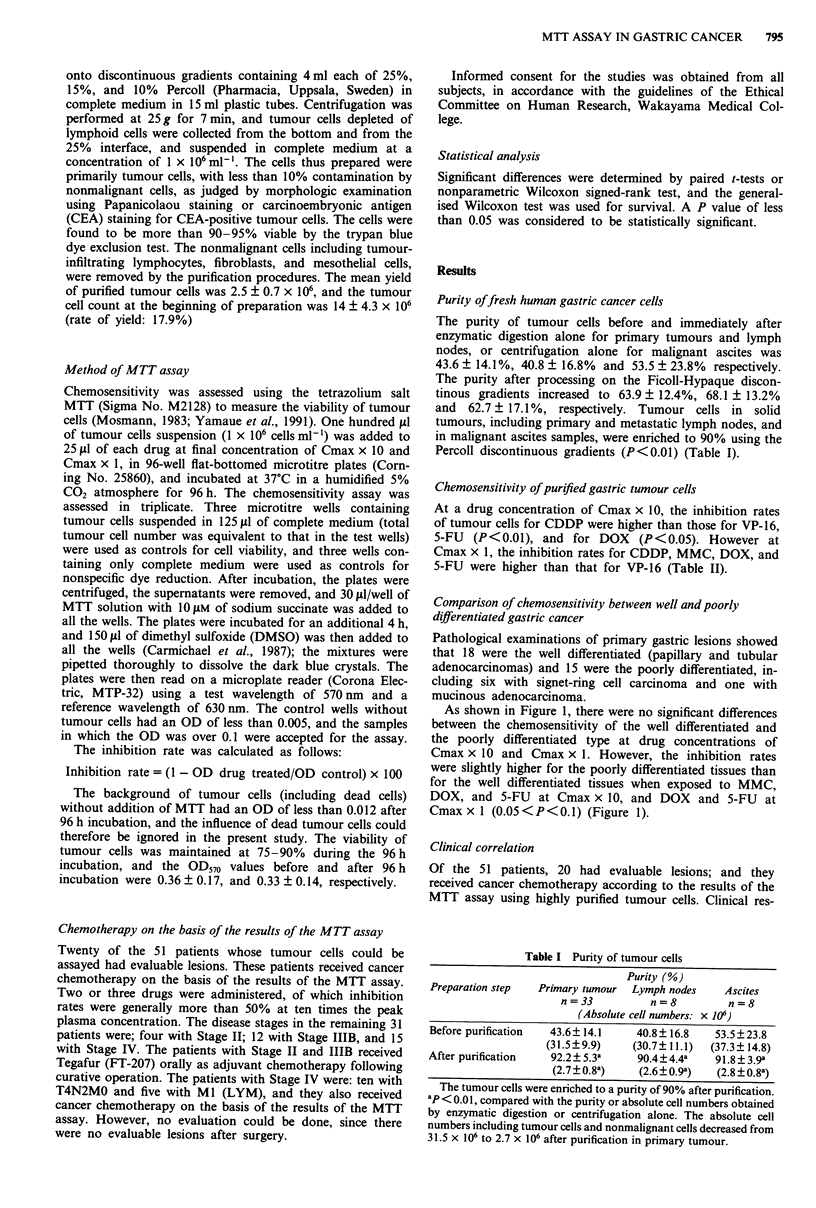

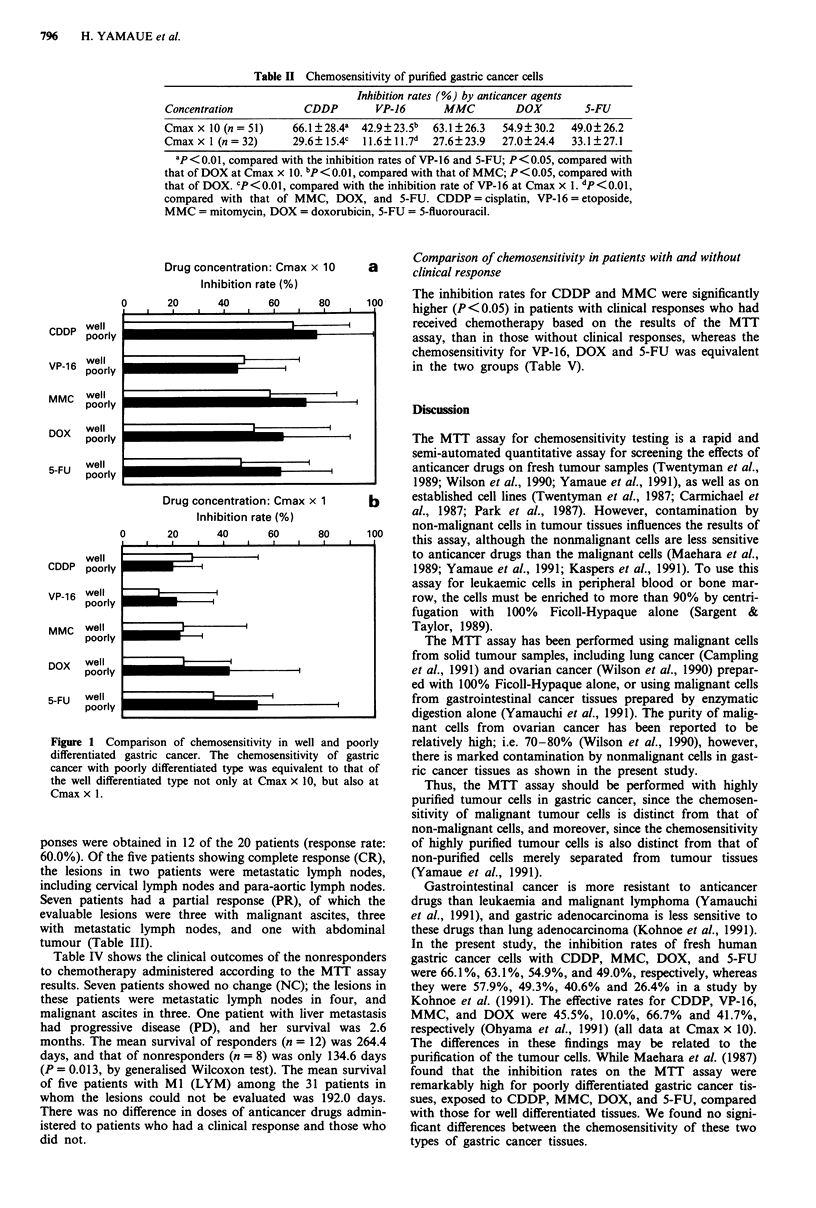

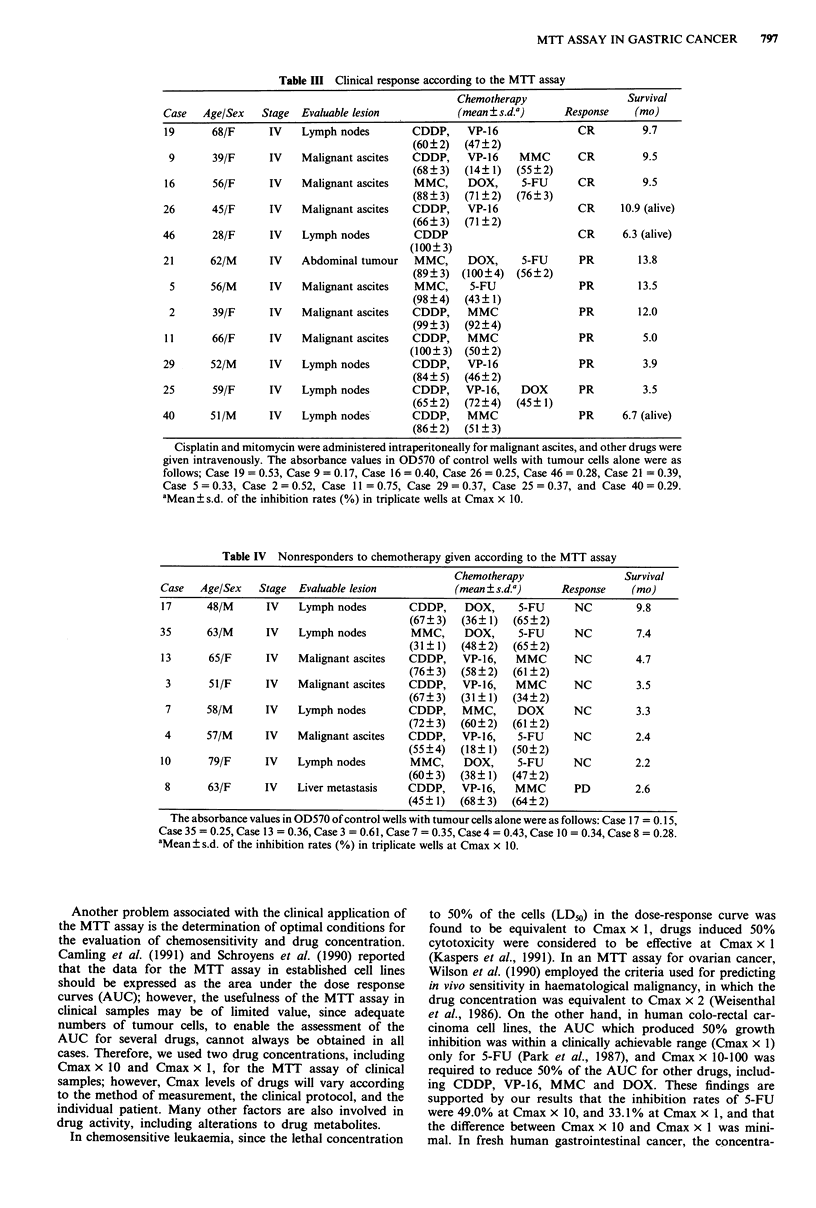

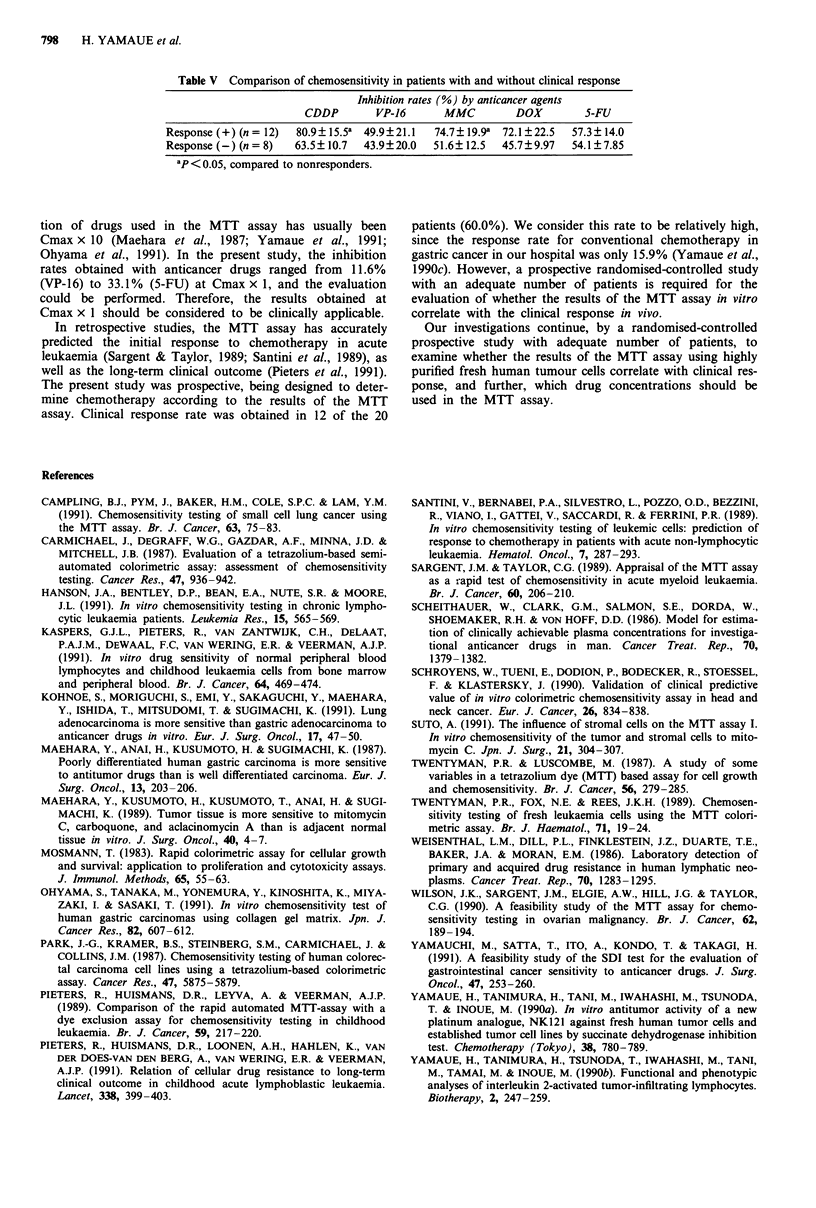

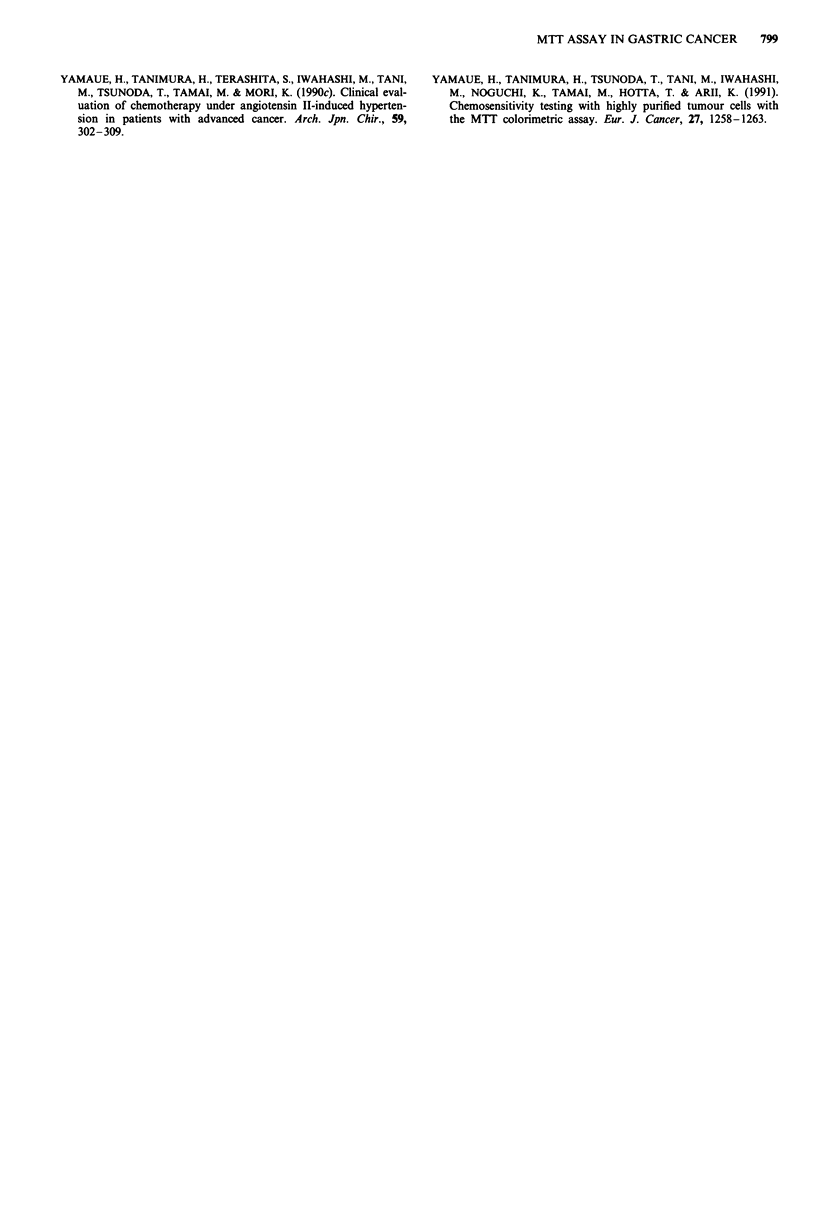


## References

[OCR_00669] Campling B. G., Pym J., Baker H. M., Cole S. P., Lam Y. M. (1991). Chemosensitivity testing of small cell lung cancer using the MTT assay.. Br J Cancer.

[OCR_00674] Carmichael J., DeGraff W. G., Gazdar A. F., Minna J. D., Mitchell J. B. (1987). Evaluation of a tetrazolium-based semiautomated colorimetric assay: assessment of chemosensitivity testing.. Cancer Res.

[OCR_00680] Hanson J. A., Bentley D. P., Bean E. A., Nute S. R., Moore J. L. (1991). In vitro chemosensitivity testing in chronic lymphocytic leukaemia patients.. Leuk Res.

[OCR_00685] Kaspers G. J., Pieters R., Van Zantwijk C. H., De Laat P. A., De Waal F. C., Van Wering E. R., Veerman A. J. (1991). In vitro drug sensitivity of normal peripheral blood lymphocytes and childhood leukaemic cells from bone marrow and peripheral blood.. Br J Cancer.

[OCR_00692] Kohnoe S., Moriguchi S., Emi Y., Sakaguchi Y., Maehara Y., Ishida T., Mitsudomi T., Sugimachi K. (1991). Lung adenocarcinoma is more sensitive than gastric adenocarcinoma to anticancer drugs in vitro.. Eur J Surg Oncol.

[OCR_00698] Maehara Y., Anai H., Kusumoto H., Sugimachi K. (1987). Poorly differentiated human gastric carcinoma is more sensitive to antitumor drugs than is well differentiated carcinoma.. Eur J Surg Oncol.

[OCR_00706] Maehara Y., Kusumoto H., Kusumoto T., Anai H., Sugimachi K. (1989). Tumor tissue is more sensitive to mitomycin C, carboquone, and aclacinomycin A than is adjacent normal tissue in vitro.. J Surg Oncol.

[OCR_00710] Mosmann T. (1983). Rapid colorimetric assay for cellular growth and survival: application to proliferation and cytotoxicity assays.. J Immunol Methods.

[OCR_00717] Ohyama S., Tanaka M., Yonemura Y., Kinoshita K., Miyazaki I., Sasaki T. (1991). In vitro chemosensitivity test of human gastric carcinomas using collagen gel matrix.. Jpn J Cancer Res.

[OCR_00721] Park J. G., Kramer B. S., Steinberg S. M., Carmichael J., Collins J. M., Minna J. D., Gazdar A. F. (1987). Chemosensitivity testing of human colorectal carcinoma cell lines using a tetrazolium-based colorimetric assay.. Cancer Res.

[OCR_00727] Pieters R., Huismans D. R., Leyva A., Veerman A. J. (1989). Comparison of the rapid automated MTT-assay with a dye exclusion assay for chemosensitivity testing in childhood leukaemia.. Br J Cancer.

[OCR_00733] Pieters R., Huismans D. R., Loonen A. H., Hählen K., van der Does-van den Berg A., van Wering E. R., Veerman A. J. (1991). Relation of cellular drug resistance to long-term clinical outcome in childhood acute lymphoblastic leukaemia.. Lancet.

[OCR_00740] Santini V., Bernabei P. A., Silvestro L., Dal Pozzo O., Bezzini R., Viano I., Gattei V., Saccardi R., Ferrini P. R. (1989). In vitro chemosensitivity testing of leukemic cells: prediction of response to chemotherapy in patients with acute non-lymphocytic leukemia.. Hematol Oncol.

[OCR_00747] Sargent J. M., Taylor C. G. (1989). Appraisal of the MTT assay as a rapid test of chemosensitivity in acute myeloid leukaemia.. Br J Cancer.

[OCR_00752] Scheithauer W., Clark G. M., Salmon S. E., Dorda W., Shoemaker R. H., Von Hoff D. D. (1986). Model for estimation of clinically achievable plasma concentrations for investigational anticancer drugs in man.. Cancer Treat Rep.

[OCR_00759] Schroyens W., Tueni E., Dodion P., Bodecker R., Stoessel F., Klastersky J. (1990). Validation of clinical predictive value of in vitro colorimetric chemosensitivity assay in head and neck cancer.. Eur J Cancer.

[OCR_00765] Suto A. (1991). The influence of stromal cells on the MTT assay (I)--In vitro chemosensitivity of the tumor and stromal cells to mitomycin C.. Jpn J Surg.

[OCR_00775] Twentyman P. R., Fox N. E., Rees J. K. (1989). Chemosensitivity testing of fresh leukaemia cells using the MTT colorimetric assay.. Br J Haematol.

[OCR_00770] Twentyman P. R., Luscombe M. (1987). A study of some variables in a tetrazolium dye (MTT) based assay for cell growth and chemosensitivity.. Br J Cancer.

[OCR_00780] Weisenthal L. M., Dill P. L., Finklestein J. Z., Duarte T. E., Baker J. A., Moran E. M. (1986). Laboratory detection of primary and acquired drug resistance in human lymphatic neoplasms.. Cancer Treat Rep.

[OCR_00786] Wilson J. K., Sargent J. M., Elgie A. W., Hill J. G., Taylor C. G. (1990). A feasibility study of the MTT assay for chemosensitivity testing in ovarian malignancy.. Br J Cancer.

[OCR_00792] Yamauchi M., Satta T., Ito A., Kondo T., Takagi H. (1991). A feasibility study of the SDI test for the evaluation of gastrointestinal cancer sensitivity to anticancer drugs.. J Surg Oncol.

[OCR_00813] Yamaue H., Tanimura H., Terashita S., Iwahashi M., Tani M., Tsunoda T., Tamai M., Mori K. (1990). Clinical evaluation of chemotherapy under angiotensin II-induced hypertension in patients with advanced cancer.. Nihon Geka Hokan.

[OCR_00805] Yamaue H., Tanimura H., Tsunoda T., Iwahashi M., Tani M., Tamai M., Inoue M. (1990). Functional and phenotypic analyses of interleukin 2-activated tumor-infiltrating lymphocytes.. Biotherapy.

[OCR_00820] Yamaue H., Tanimura H., Tsunoda T., Tani M., Iwahashi M., Noguchi K., Tamai M., Hotta T., Arii K. (1991). Chemosensitivity testing with highly purified fresh human tumour cells with the MTT colorimetric assay.. Eur J Cancer.

